# Hydrogen sulphide induces μ opioid receptor-dependent analgesia in a rodent model of visceral pain

**DOI:** 10.1186/1744-8069-6-36

**Published:** 2010-06-11

**Authors:** Eleonora Distrutti, Sabrina Cipriani, Barbara Renga, Andrea Mencarelli, Marco Migliorati, Stefano Cianetti, Stefano Fiorucci

**Affiliations:** 1 S.C. di Gastroenterologia, Azienda Ospedaliera di Perugia, Perugia, Italia; 2 Dipartimento di Medicina Clinica e Sperimentale, Università degli Studi di Perugia, Perugia, Italia; 3 Dipartimento di Scienze Chirurgiche, Radiologiche e Odontostomatologiche, Università degli Studi di Perugia, Perugia, Italia

## Abstract

**Background:**

Hydrogen sulphide (H_2_S) is a gaseous neuro-mediator that exerts analgesic effects in rodent models of visceral pain by activating K_ATP _channels. A body of evidence support the notion that K_ATP _channels interact with endogenous opioids. Whether H_2_S-induced analgesia involves opioid receptors is unknown.

**Methods:**

The perception of painful sensation induced by colorectal distension (CRD) in conscious rats was measured by assessing the abdominal withdrawal reflex. The contribution of opioid receptors to H_2_S-induced analgesia was investigated by administering rats with selective μ, κ and δ opioid receptor antagonists and antisenses. To investigate whether H_2_S causes μ opioid receptor (MOR) transactivation, the neuronal like cells SKNMCs were challenged with H_2_S in the presence of MOR agonist (DAMGO) or antagonist (CTAP). MOR activation and phosphorylation, its association to β arrestin and internalization were measured.

**Results:**

H_2_S exerted a potent analgesic effects on CRD-induced pain. H_2_S-induced analgesia required the activation of the opioid system. By pharmacological and molecular analyses, a robust inhibition of H_2_S-induced analgesia was observed in response to central administration of CTAP and MOR antisense, while κ and δ receptors were less involved. H_2_S caused MOR transactivation and internalization in SKNMCs by a mechanism that required AKT phosphorylation. MOR transactivation was inhibited by LY294002, a PI3K inhibitor, and glibenclamide, a K_ATP _channels blocker.

**Conclusions:**

This study provides pharmacological and molecular evidence that antinociception exerted by H_2_S in a rodent model of visceral pain is modulated by the transactivation of MOR. This observation provides support for development of new pharmacological approaches to visceral pain.

## Introduction

Visceral pain is the most common sign of acute and chronic gastrointestinal, pelvic and genitourinary diseases. As one of the most common causes of persistent disability, visceral pain represents a frequent reason for patients to seek medical treatment. Despite multiple therapeutic approaches, the treatment of visceral pain remains a significant challenge.

A complex network of signaling molecules mediates perception of visceral pain [1]. Hydrogen sulphide (H_2_S) is a gaseous neuromodulator generated from L-cysteine by the activity of two pyrodoxal-5'-phosphate-dependent enzymes, the cystathionine γ-lyase (CSE) and the cystathionine β-synthase (CBS) [2-5], that exerts regulatory activities in the gastrointestinal tract [1,4]. In the central nervous system H_2_S mediates the induction of hippocampal long-term potentiation [6-8] and the release of the corticotropin releasing hormone from the hypothalamus [9], enhances NMDA receptor-mediated responses [8] and protects against peroxynitrite-induced neuronal toxicity [10]. ATP-sensitive potassium (K_ATP_) channels have been identified as important mediators of several effects exerted by H_2_S [2,3,10]. Thus, glibenclamide, a K_ATP _channels blocker, attenuates analgesic effect of H_2_S in a model of visceral pain induced by colorectal distension (CRD) in healthy and post-colitis, allodynic rats [11,12].

Opioid receptors are G protein-coupled receptors (GPCRs) and the main receptors involved in the modulation of pain in mammals [13,14]. The principal opioid receptor subtypes, μ (MOR), δ (DOR) and κ (KOR), are all expressed in the spinal cord and in the brain contributing to the modulation of nociceptive transmission. In addition, the μ and κ opioid receptors are also expressed in the enteric nervous system. MOR is the preferred receptor for potent analgesics with high potential for abuse, such as morphine [14]. Endogenous opioids, including enkephalins, endorphins and opiates like etorphine, induce rapid μ receptor endocytosis in neurons and transfected cells [15,16], a process called internalization that is widely used as a marker of MOR activation [17,18].

Opioid receptors and K_ATP _channels converge in regulating release of neurotransmitters, smooth muscle contractions and neuronal excitability with both signaling pathways being effective in attenuating perception of visceral painful sensations in animal models and patients [19,20]. Whether H_2_S signaling integrates with the opioid system, however, is still unknown.

In the present study we provide evidence that antinociception exerted by H_2_S in a rodent model of visceral pain is selectively modulated by the intervention of μ opioid receptors. By *in vitro *studies we demonstrated that a previously unrecognized neuronal circuit with H_2_S-activated K_ATP _channels transactivating the μ opioid receptor supports the analgesic activities of H_2_S. These results identify new pharmacological targets in the treatment of chronic visceral pain.

## Results

### H_2_S inhibits CRD-induced nociception

In all experimental settings two sequential distension-effect curves were constructed. The first distension-effect curve was used as a control, while the second was constructed in response to saline or specified drug. In all experiments animals were awake and no changes in the consciousness state were produced by Na_2_S administration.

**Figure 1:**
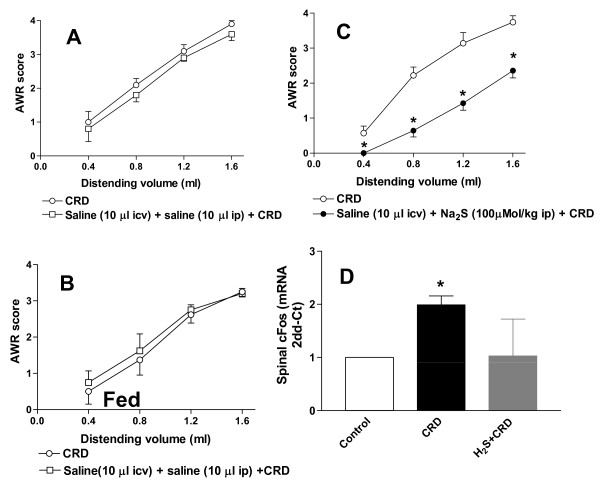
**Na_2_S induces antinociception**. CRD induces a volume-dependent increase of the AWR score in both fasting and fed rats (panels A and B respectively) and Na_2_S (100 μMol/kg i.p.) causes a significant reduction of visceral sensitivity and pain (panel C). Data are mean ± SEM of 5 rats. *p < 0.05 versus CRD. CRD induces the increase of spinal cFos expression that is downregulated by Na_2_S (panel D). Data are mean ± SEM of 5 rats. *p < 0.05 versus control.

CRD (0.4-1.6 ml water) elicited a volume-dependent increase of the AWR scores which was rapid in onset, persisted for the duration of the distension period (Figure 1, panel A) and returned to the baseline immediately after the distension was stopped. In the fed animals CRD elicited a similar pattern of response (Figure 1, panel B). Injected intraperitoneally (i.p.) at the dose of 100 μMol/kg, Na_2_S decreased the AWR score (Figure 1, panel C, p < 0.05 versus CRD alone) and determined a significant increase of colorectal compliance (data not shown) indicating that H_2_S induced a myorelaxant action on colonic smooth muscle cells. The antinociceptive effect of Na_2_S was confirmed by analysis of spinal cFos mRNA expression. Thus, Na_2_S administration abrogated cFos mRNA expression induced in the spinal cord by CRD (Figure 1, panel D, p < 0.05 versus control).

### μ opioid receptors antagonism inhibits the H_2_S-induced antinociception

**Figure 2:**
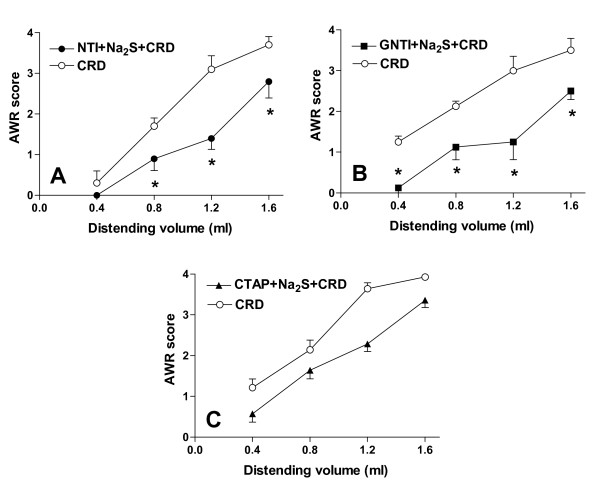
**CTAP reverses the Na_2_S-induced antinociception**. Pre-treating rats with the selective μ opioid receptor antagonist CTAP (0.09 mg/kg i.c.v. thirty minutes before Na_2_S; panel C) abrogates the antinociceptive effect of Na_2_S (100 μMol/kg i.p.). In contrast, the selective δ opioid receptor antagonist NTI (4 μg/kg i.c.v. five minutes before Na_2_S, panel A) and the selective κ opioid receptor antagonist GNTI (0.08 μmg/kg i.c.v. three days before Na_2_S, panel B) do not inhibit the analgesic effect of Na_2_S, indicating that δ and κ opioid receptors have no effects on Na_2_S-induced antinociception. Data are mean ± SEM of 5 rats. *p < 0.05 versus CRD.

**Figure 3:**
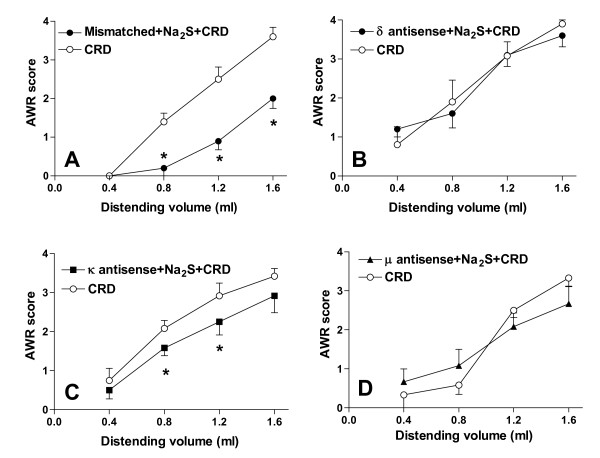
**The selective antisense oligodeoxynucleotide probes against DOR and MOR reverse the Na_2_S-induced antinociception**. Pre-treating rats with both the mismatched antisense oligodeoxynucleotides (panel A) and the κ opioid receptor antisense oligodeoxynucleotides (panel C) does not modify the H_2_S-induced decrease of the AWR score, confirming that KOR does not cause any change on the Na_2_S-induced analgesia. In contrast, oligodeoxynucleotide probes against DOR and MOR reverse the antinociception caused by Na_2_S (panel B and D respectively). Data are mean ± SEM of 5 rats. *p < 0.05 versus CRD.

The antinociceptive effect of Na_2_S on CRD-induced pain was studied by pre-treating animals with selective opioid receptor antagonists. As illustrated in Figure 2, while the DOR antagonist NTI, and the KOR antagonist GNTI injected intracerebroventricularly (i.c.v.) had no effect on Na_2_S-induced antinociception (Figure 2, panels A and B respectively, p < 0.05 versus CRD), the selective MOR antagonist CTAP injected i.c.v. reverted analgesia induced by Na_2_S (Figure 2, panel C) without interfering with its myorelaxant activity (data not shown). Administering rats with NTI, GNTI and CTAP alone had no effect on CRD-induced nociception (data not shown). To confirm the above mentioned results by another method, we injected rats i.c.v. with oligodeoxynucleotide antisenses directed against each specific opioid receptor subtype. While pre-treating rats with mismatched antisenses failed to modulate Na_2_S-induced analgesia (Figure 3, panel A, p < 0.05 versus CRD), the analgesic activity of H_2_S on CRD-induced pain was abrogated by pre-treating animals with δ and μ opioid receptor antisenses (Figures 3, panels B and D respectively). In contrast, no effect was observed with the κ opioid receptor antisense (Figure 3, panel C, p < 0.05 versus CRD). All antisenses had no effect on colonic myorelaxation induced by Na_2_S (data not shown). Finally, administering rats with antisenses alone had no effect on nociception induced by CRD (data not shown).

**Figure 4:**
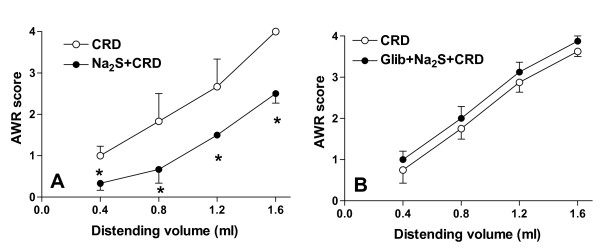
**Glibenclamide reverses the Na_2_S-induced antinociception**. In a different experiment we have analyzed the role of the K_ATP _channels on the H_2_S-induced analgesia (panel A). Pre-treating rats with the K_ATP _channels selective blocker glibenclamide (2.8 μmol/kg i.v.) completely reverses the Na_2_S-induced analgesia (panel B) without any effects on the change of the colonic compliance induced by Na_2_S. Data are mean ± SEM of 5 rats. *p < 0.05 versus CRD.

To determine whether the analgesic effect of Na_2_S was modulated by K_ATP _channels, we performed an experiment by using the K_ATP _channel antagonist glibenclamide. The antinociceptive effect of Na_2_S (Figure 4, panel A) was reverted by blocking the K_ATP _channels with glibenclamide (Figure 4, panel B), while treating rats with glibenclamide alone failed to modulate nociception induced by CRD (data not shown).

### H_2_S induces MOR activation and internalization

**Figure 5:**
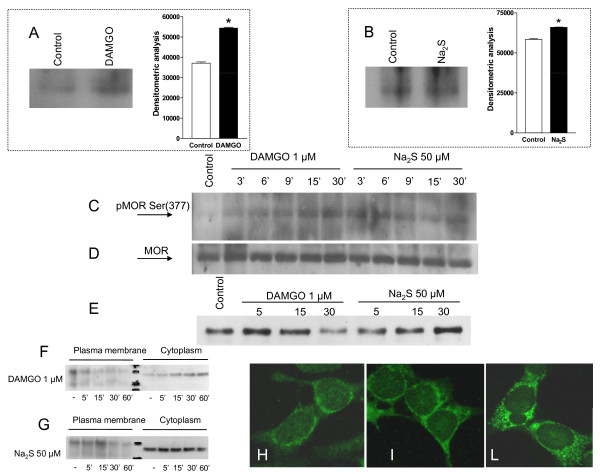
**Na_2_S induces MOR activation and phosphorylation, the recruitment of β arrestin and MOR internalization**. Both DAMGO (1 μM) and Na_2_S (50 μM) induce MOR activation (panel A and B respectively). Treating SKNMCs with both DAMGO and Na_2_S results in MOR phosphorylation that is time-dependent. DAMGO induces MOR phosphorylation at Ser(377) that is maximal at 30 minutes and, similarly, H_2_S induces MOR phosphorylation that peaks at 3-6 minutes and persists until 30 minutes (panel C). The total DAMGO-induced and H_2_S-induced MOR phosphorylation is unchanged within the duration of the experiment (panel D). Co-immunoprecipitation experiments demonstrate that DAMGO induces the rapid complex between MOR and β arrestin with the peak at 5-15 minutes and, similarly, H_2_S induces the co-immunoprecipitation of MOR and β arrestin that peaked at 30 minutes (panel E), indicating that H_2_S induces the interaction between β arrestin and MOR. At the cell membrane fractioning experiments, DAMGO (1 μM) causes the disappearance of MOR from the plasma membrane fraction at 5 minutes and this effect is maximal at 60 minutes. At the same time there is a progressive increment of MOR presence in the cytoplasmatic fraction (panel F). After Na_2_S (50 μM), MOR disappears from the plasma membrane fraction at 30 minutes with the maximal effect at 60 minutes and, in contemporary, it passes into the cytoplasmatic fraction (panel G). At the confocal microscopy SKNMCs exhibit MOR immunoreactivity predominantly localized at the cell surface in nonstimulated condition (panel H) and it translocates to cytoplasm after activation with DAMGO (panel I), which is known to induce MOR internalization. Na_2_S induces a massive translocation of MOR from plasma membrane into the cytoplasm in most neurons (panel L). Data are representative of at least 3 experiments. *p < 0.05 vs control.

To investigate the mechanisms by which Na_2_S activates MOR, experiments were carried out in SKNMC cells, a neuron-like cell line that expresses functional μ opiod receptors. Agonist-induced activation of MOR results in conformational changes of the extracellular portion of the receptor that unmasks a specific epitope near to the N-terminus. By using a specific antibody that target this epitope, we have investigated whether Na_2_S causes MOR activation. As illustrated in Figure 5, panels A and B, MOR activation was detected in cells exposed to either the μ receptor-selective enkephalin analog DAMGO and Na_2_S, indicating that exposure to Na_2_S induced an activity-dependent conformational change of the N-terminal region of the MOR. Further, exposure of SKNMCs to Na_2_S caused the direct phosphorylation of MOR in the Ser(377) (Figure 5, panel C), a measure of the receptor activation, and exposure of cells to DAMGO also caused a robust induction of MOR phosphorylation in the serine residue, thought that the kinetic of the two effects was different (Figure 5, panel C). As expression of total MOR protein did not change (Figure 5, panel D), these results demonstrated that exposure of SKNMCs to Na_2_S induced a rapid and persistent phosphorylation of the μ opioid receptor in a site that is functionally linked to its activation.

Following its activation, MOR is rapidly internalized after its recruitment into a multiprotein complex with β arrestin. By co-immunoprecipitation experiments (Figure 5, panel E) we found that exposure of SKNMCs to DAMGO and Na_2_S caused a robust induction of MOR association with β arrestin. By membrane fraction technique we found that DAMGO caused MOR internalization as shown by its disappearance from the plasma membrane and relocation into the cytosol fraction as early as 5 minutes of exposure (Figure 5, panel F). A similar pattern was observed in response to Na_2_S, thought the time course was slightly different (Figure 5, panel G). These findings were confirmed by confocal microscopy analysis (Figure 5, panels H-L). Thus, while resting SKNMCs exhibited MOR immunoreactivity predominantly at the cell surface (Figure 5, panel H), a massive translocation of receptor to the cytosol occurred in cells exposed to DAMGO (Figure 5, panel I) and Na_2_S (Figure 5, panel L).

**Figure 6:**
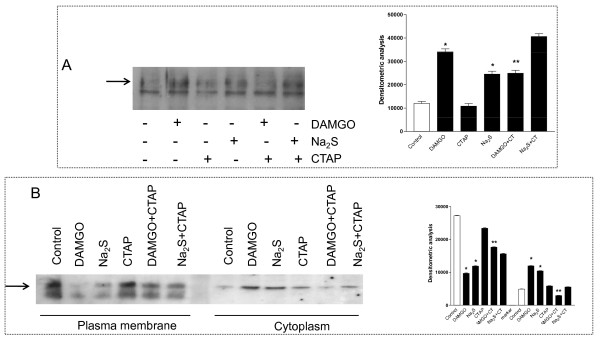
**CTAP only partially inhibits the Na_2_S-induced MOR internalization**. SKNMCs are stimulated with DAMGO (1 μM) or Na_2_S (50 μM) in presence or in absence of CTAP (1 μM) and the effects on MOR activation and internalization are detected. CTAP inhibits the MOR activation induced by DAMGO while it has no effect on that induced by Na_2_S (panel A). CTAP blocks the DAMGO-induced MOR internalization but, in contrast, it only partially inhibits the Na_2_S-induced MOR internalization (panel B). Data are representative of at least 3 experiments. *p < 0.05 vs control, **p < 0.05 vs DAMGO alone.

To further investigate whether activation of MOR by Na_2_S occurs by direct receptor activation or is mediated by receptor transactivation, we challenged SKNMCs with the highly selective μ receptor antagonist CTAP. Results from these experiments demonstrate that while MOR activation induced by DAMGO was abrogated by CTAP, the antagonist had no effects on MOR activation induced by Na_2_S (Figure 6, panel A). Similarly, CTAP was effective in preventing MOR internalization induced by DAMGO but only partially prevented cytosolic MOR translocation induced by Na_2_S treatment (Figure 6, panel B).

### H_2_S induces PI3K/AKT activation

**Figure 7:**
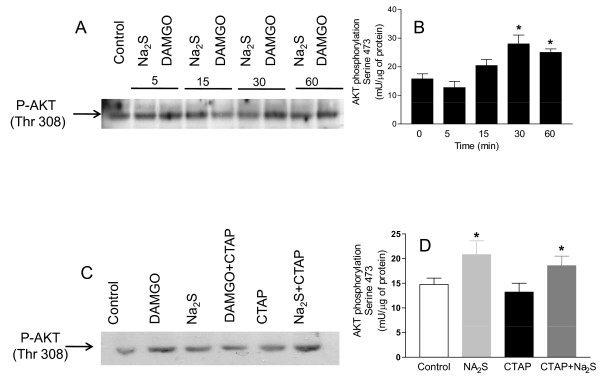
**Na_2_S induces AKT phosphorylation**. Exposure to both DAMGO (1 μM) and Na_2_S (50 μM) causes AKT phosphorylation on Threonine 308 as detected by Western blot analysis (panel A). Moreover, Na_2_S induces AKT phosphorylation on Ser(473) as detected by phospho AKT assay (panel B). CTAP inhibits the DAMGO-induced AKT phosphorylation on Thre(308) (panel C), while it does not prevent that induced by Na_2_S on Thre(308) and Ser(473) (panels C and D). Data are representative of at least 3 experiments. Data on AKT phosphorylation are mean ± SE of 5 experiments. *p < 0.05 vs control.

Because H_2_S induces AKT phosphorylation [21] and AKT is also activated in response to MOR activation by DAMGO [22], we have investigated whether Na_2_S induces AKT phosphorylation in SKNMCs. Results of these experiments demonstrated that both DAMGO and Na_2_S caused a long-lasting phosphorylation of AKT in Threonine 308 (Thre308), a marker of AKT activation (Figure 7, panel A). The induction of AKT phosphorylation by Na_2_S was time dependent as further confirmed by an immunoassay that specifically detects AKT phosphorylation on Serine 473 (Ser473) (Figure 7, panel B). AKT phosphorylation induced by DAMGO was reversed by CTAP (Figure 7C). However, CTAP failed to inhibit AKT phosphorylation induced by Na_2_S (Figure 7, panel C and D, p < 0.05 versus control).

**Figure 8:**
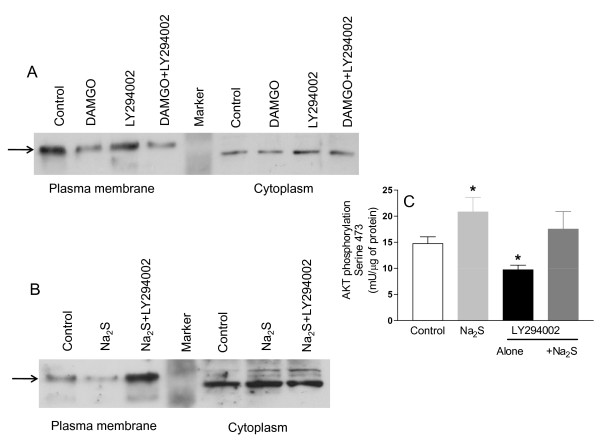
**LY294002 inhibits the Na_2_S-induced MOR internalization and AKT phosphorylation**. The selective PI3K inhibitor LY294002 has no effects on DAMGO-induced MOR internalization (panel A), while it blocks that induced by Na_2_S (panel B). Furthermore, LY294002 inhibits the AKT phosphorylation induced by Na_2_S on Ser(473) (panel C). Data are representative of at least 3 experiments. Data on AKT phosphorylation are mean ± SE of 5 experiments. *p < 0.05 vs control.

To investigate the role of the PI3K/AKT pathway in Na_2_S-induced MOR internalization, SKNMCs were pre-treated with the selective PI3K inhibitor LY294002 (50 μM). LY294002 had no effect on DAMGO-induced MOR internalization (Figure 8, panel A), but prevented MOR internalization induced by Na_2_S (Figure 8, panel B). Moreover, LY294002 abrogated AKT phosphorylation induced by Na_2_S (Figure 8, panel C).

### SKNMCs express K_ATP _channels subunits: glibenclamide inhibits MOR activation and AKT phosphorylation

**Figure 9:**
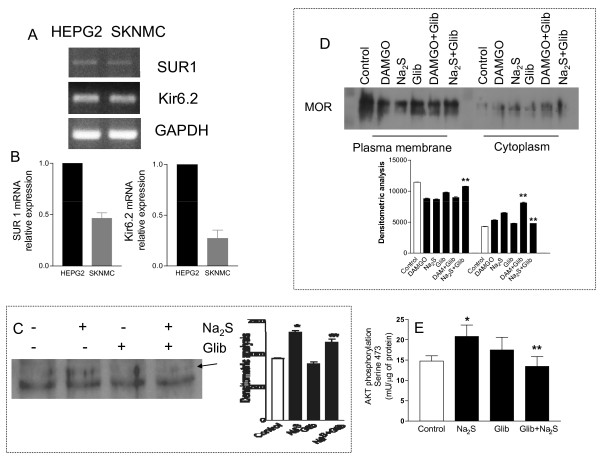
**Glibenclamide inhibits Na_2_S-induced MOR activation and internalization and AKT phosphorylation**. Qualitative PCR (panel A) and Quantitative Real-Time PCR (panel B) showing the expression of Kir6.2 and SUR1 in HepG2 (positive control) demonstrate that SKNMCs express both the K_ATP _channels subunits Kir6.2 and SUR1. SKNMCs are stimulated with Na_2_S (50 μM) in presence or in absence of glibenclamide (1 μM) for 60 minutes. Glibenclamide prevents the Na_2_S-induced MOR activation (panel C) and internalization (panel D), while it has no effect on MOR internalization induced by DAMGO (panel D). Finally, glibenclamide inhibits AKT phosphorylation induced by Na_2_S, as assessed by phospho-immunoassay (panel F). Data are representative of at least 3 experiments. Data on AKT phosphorylation are mean ± SE of 5 experiments. *p < 0.05 vs control; **p < 0.05 vs DAMGO or Na_2_S alone.

Because glibenclamide abrogates analgesia induced by Na_2_S suggesting the involvement of K_ATP _channels, we have investigated whether SKNMCs express functional K_ATP _channels. By RT-PCR we found that both the Kir6.2 and SUR1 subunits were expressed in the SKNMCs (Figure 9, panels A and B respectively) and by antagonism experiments we demonstrated that these channels were functionally active because glibenclamide (1 μM) inhibited MOR activation (Figure 9, panel C), MOR internalization (Figure 9, panel D) and AKT phosphorylation (Figure 9, panel E) induced by Na_2_S.

## Discussion

In this study we have demonstrated that H_2_S induces μ opioid-dependent analgesia in a rodent models of visceral pain. Moreover, in a supplementary experiment, we have demonstrated that, in contrast to what previously reported on the effect of meal on visceral perception in humans [23-25], CRD induces a similar painful response in both fasting and fed animals, indicating that meal has no influence on visceral perception in this experimental setting. However, more experiments are needed to clarify this particular issue.

Several mechanisms might explain the antinociceptive effect of H_2_S. *First*, a bluntness of sensorial functions that mimics a pain-free condition is unlikely because we did not observe any change in the consciousness of the rats during these studies. *Second*, as H_2_S causes a relaxation of smooth muscle cells, H_2_S could simply act as myorelaxant agent. However, this explanation seems unlikely, given that we have previously demonstrated that H_2_S inhibited CRD-induced nociception at doses that did not modify the colorectal compliance [11]. A *third*, more likely explanation would be that the antinociceptive effect of H_2_S is mediated by a direct inhibitory activity on colorectal afferent pathways. Consistent with this view, we found that administration of H_2_S decreased spinal cord expression of cFos mRNA.

The widespread occurrence of the opioid receptors indicates that opioids have the potential for affecting multiple systems, including nervous, hormonal and immunological systems. Opioid receptors have specific pharmacological profiles and physiological functions, maintain a certain degree of selectivity for various opioid ligands, and display unique patterns of expression in the nervous system, even though there is overlap in their binding affinity, distribution and function [26,27]. Agonists of μ opioid receptors produce analgesia, affect mood and rewarding behavior and alter respiratory, cardiovascular, gastrointestinal and neuroendocrine functions [27]. While the actions of μ opioid agonists are invariably analgesic, those of κ agonists can be either analgesic or antianalgesic, the last effect being mediated by a functional antagonism on the action of μ receptor agonists. δ opioid receptor agonists also are potent analgesics in animals and, in isolated cases, have proved useful in human beings [27]. The main barrier to the clinical use of δ agonists is that the most available agents are peptides that do not cross the blood-brain barrier, thus requiring intraspinal administration. The ability to elucidate the roles of opioid receptor subtypes in the mediation of analgesia was first enhanced by the development of selective opioid receptor subtype antagonists direct against μ, κ and δ receptors and subsequently by the use of antisense probes to establish the relationship of the cloned receptors to opioid actions using sequences complementary to regions of specific exons of mRNA to down-regulate opioid receptor proteins.

In the present study we described for the first time that the analgesic effects of H_2_S is reverted by central opioid antagonism. In particular, the selective μ antagonist CTAP, centrally administered, inhibits the H_2_S-induced analgesia while the selective κ and δ receptor antagonists have no effect. Moreover, when the selective, centrally administered antisense olygodeoxynucleotides have been used, the antisense oligodeoxynucleotides direct against μ receptors confirm the pharmacological data, suggesting that the μ opioid receptors are primarily involved in the mediation of H_2_S-induced analgesia. In contrast, our pharmacological and antisense oligodeoxynucleotides studies converge onto the indication that κ opioids receptors do not alter the H_2_S-mediated effects on visceral sensitivity and pain. Previous pharmacological data indicating that activation of δ opioid receptors attenuates responses to noxious stimuli [28-31] were confirmed by studies conducted by using olygodeoxynucleotide probes direct against δ opioids receptors [32-34]. In our study, the selective δ opioid receptor antagonist NTI has no effect on the H_2_S-induced analgesia, while the oligodeoxynucleotide probes against DOR cause the reversion of the analgesic effect exerted by H_2_S, suggesting a relatively minor contribution of δ opioid receptors to pain modulation by H_2_S. However, the discrepancy between pharmacological and antisense data about the modulation of H_2_S-induced analgesia by δ opioid receptors needs to be clarified by further studies.

Although hundreds of studies performed by using both pharmacological approaches and antisense probes focused on the different ability of the opioid receptors to cause analgesia, our data fit with the notion that MOR is identified as the most important opioid receptor linked with pain system so that the selective μ endogenous or exogenous agonists are invariably analgesic while selective μ opioid antagonists induce or exacerbate pain by blocking the effects of μ agonists in several experimental conditions. Because antisenses are highly selective and specific in downregulating one opioid receptor without interfering with the activity of other subtypes [35], these pharmacological and antisense studies converge in the indication that μ opioid receptors mediate H_2_S-induced analgesia.

In the present study we have provided evidence that the analgesic activity of H_2_S is mediated by the recruitment of μ opioid receptor. In addition to specific pharmacological antagonism exerted *in vivo *by CTAP and MOR antisense on antinociceptive activity of H_2_S, results from *in vitro *pharmacological dissection of signaling pathways activated by H_2_S are consistent in supporting the view that H_2_S transactivates the μ opioid receptor. Exposure of SKNMCs to H_2_S causes conformational changes of the extracellular tail of MOR that are known to be associated with an activated state of the receptor. These conformational changes of the N-terminus unmasks a specific epitope that can be detected by an activation-state specific antibody [36,37]. Results of experiments carried out using this approach have revealed that exposure of SKNMCs to H_2_S causes a change in the conformational status of MOR similar to that induced by the enkephalin analog DAMGO, a potent agonist of MOR. Further, and similarly to DAMGO, H_2_S causes a robust, time- and concentration-dependent phosphorylation of MOR in Ser(377), a site that is specifically required to induce receptor activation and internalization by DAMGO. Previous studies have shown that among the 12° potential phosphorylation sites present in the C-tail of MOR, only Ser(363), Thre(370) and Ser(375) are involved in MOR phosphorylation and linked to receptor activation [38]. DAMGO-induced MOR phosphorylation occurs at Thre (370) and Ser(375) [Ser(377) in human receptor] but only mutation of Ser(375) is reported to attenuate the rate and extent of receptor internalization [38].

One important observation we made is that phosphorylation of MOR's Ser(377) induced by H_2_S is rapidly reversible. Because prolonged activation of μ opioid receptors leads to their phosphorylation, internalization, desensitization and down-regulation and represents one the main biochemical substrates of morphine tolerance, the fact that H_2_S causes a short-lasting receptor phosphorylation and that rapid receptor phosphorylation (min) does not directly correlate with the relatively slow rate of desensitization (h) of MOR induced by morphine [27], suggests that this mediator is unlikely to play a role in long term desensitization of MOR and could still be a pharmacological target in situation of MOR desensitization

Mutational analysis has demonstrated that phosphorylation of Ser (375) or Ser(377) in the human receptor is critical for DAMGO-induced MOR internalization [38]. In the present study we have shown that exposure of SKNMCs to H_2_S not only results in Ser(377) phosphorylation but also in MOR internalization. Similarly to DAMGO, H_2_S induces a loss of cell surface expression of MOR as monitored by confocal microscopy and cell membrane fractioning technique. MOR internalization induced by H_2_S is mediated by its recruitment to a protein-protein complex with β arrestin [18]. Previous studies have shown that once phosphoryled, the opioid receptor binds to β arrestin and is trafficked to clathrin-coated pits where it can subsequently be internalized into endosomes. Once internalized, endosomes containing receptors can be fused with lysosomes where receptors are proteolytically degraded or, alternatively, the receptors are dephosphoryled, resensitized and recycled back to membrane [39]. One of the main findings of the present study is that H_2_S reproduces the same effects of DAMGO in terms of MOR phosphorylation, association with β arrestin and internalization. However, H_2_S induces a slower β arrestin recruitment and MOR internalization than DAMGO, providing evidence that it does not behave as a direct MOR agonist.

Results form mechanistic studies aimed at dissecting intracellular signals activated by H_2_S in SKNMCs have shown that H_2_S activates the PI3K/AKT pathway and induces AKT phosphorylation [21]. PI3K is a lipid kinase acting as a membrane-embedded second messenger [40] and AKT is a downstream target of the PI3K [41]. Activation of MOR by DAMGO induces AKT phosphorylation [42]. Our study confirms these observations and extend this effect to H_2_S. However, while CTAP reverses AKT phosphorylation induced by DAMGO, it fails to inhibit the effects exerted by H_2_S on AKT, indicating that, despite MOR trans-activation, H_2_S-induced AKT phosphorylation is due to a direct effect of the gas on the PI3K/AKT pathway. The fact that inhibition of AKT phosphorylation by the PI3K inhibitor LY294002 prevents MOR internalization induced by H_2_S but not by DAMGO, indicates that H_2_S directly activates the PI3K/AKT pathway and that activation of this pathway is hierarchically higher in the mechanism that leads to MOR activation by H_2_S. These findings are consistent with the observation that activation and internalization of a GPCR can be regulated by activation of the PI3K/AKT pathway [43].

**Figure 10:**
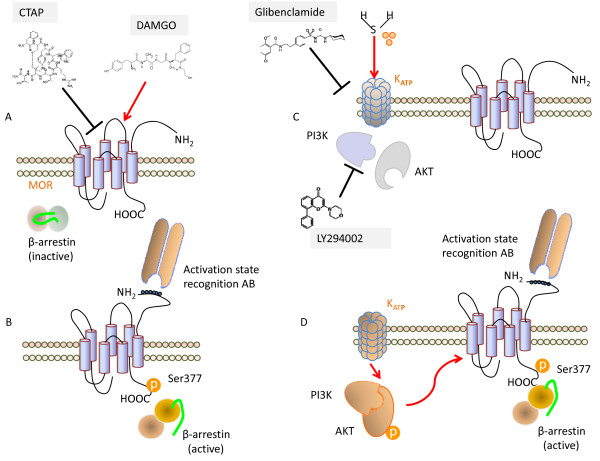
**Schematic representation of H_2_S and opioid receptor interaction**. The selective μ opioid receptor enkephalin analog DAMGO acts as a direct agonist of MOR leading to its activation, phosphorylation on Ser(377), co-immunoprecipitation with β arrestin and internalization (panel A and B). The selective MOR antagonist CTAP blocks the effects induced by DAMGO, while it only partially inhibits those induces by H_2_S. In contrast, H_2_S opens the K_ATP _channels that activate the PI3K/AKT pathway leading to MOR activation, phosphorylation, co-immunoprecipitation with β arrestin and internalization, as the selective K_ATP _channels blocker glibenclamide and the selective PI3K inhibitor LY294002 inhibit these effects (panel C and D).

The mechanism through which H_2_S targets the PI3K/AKT pathway involves K_ATP _channels. Thus not only SKNMCs express SUR1 and Kir6.2, but blocking these channels with glibenclamide abrogates AKT phosphorylation and MOR activation and internalization induced by H_2_S. This suggest a hierarchic order in the observed effects with H_2_S acting as a K_ATP _channels opener leading to activation of PI3K/AKT pathway and MOR activation and phosphorylation (Figure 10). Similar transactivation of opioid receptors by epidermal growth factor receptor has been recently described [44], however this is the first evidence of transactivation of MOR by activation of K_ATP _channels.

## Conclusion

This study demonstrates that, in a rodent model of visceral pain, H_2_S-induced analgesia is mediated by μ opioids receptor activation as, *in vivo*, the selective antagonism of MOR by i.c.v. administration of both CTAP and antisenses direct against MOR reverses the analgesic effects of H_2_S. Moreover, pre-treating rats with the K_ATP _channels selective blocker glibenclamide reverses the H_2_S-induced analgesia. The *in vitro *studies performed comparing the effect of the μ receptor-selective enkephalin analog DAMGO and H_2_S confirm these data demonstrating that, in the neuronal-cell line SKNMC, both DAMGO and H_2_S induce MOR activation and phosphorylation leading to interaction between MOR and β arrestin and MOR internalization. CTAP completely blocks MOR internalization induced by DAMGO while, in contrast, it partially inhibits MOR internalization induced by hydrogen sulphide. In addition, exposure to hydrogen sulphide causes the PI3K/AKT pathway activation and induces AKT phosphorylation. The selective PI3K inhibitor LY294002 does not interfere with the DAMGO-induced MOR internalization, while it causes the inhibition of the translocation process of MOR from the plasma membrane to the cytoplasm induced by hydrogen sulphide as well as AKT phosphorylation induced by hydrogen sulphide. As glibenclamide reverted the analgesia induced by hydrogen sulphide, we hypothize that the ATP potassium channels could modulate MOR activation induced by hydrogen sulphide. First we have demonstrated that SKNMCs express the ATP potassium channels subunits Kir6.2 and SUR1. Moreover, glibenclamide inhibits both MOR and AKT phosphorylation induced by hydrogen sulphide, demonstrating that activation of ATP potassium channels by hydrogen sulphide is a key process of these effects. On these basis we can speculate that hydrogen sulphide acts on the ATP potassium channels that induce the PI3K/AKT pathway that, on turn causes MOR activation and internalization (Figure 10). This study provides the first evidence for a cross-talk between H_2_S and the μ opioid receptors and paves the way to development of new therapeutic approaches to visceral pain.

## Methods

### Materials

Sodium sulphide (Na_2_S) was used as donor of hydrogen sulphide and was from Sigma-Aldrich (S. Louis, MO, USA). Methylene blue, glibenclamide, naltrindole (NTI) 5'-guanidinonaltrindole (GNTI),_D_-Phe-Cys-Tyr-_D_-Trp-Arg-Thr-Pen-Thr-NH_2 _(CTAP), mismatched and specific antisense olygodeoxynucleotide probes for opioid receptors, [_D_-Ala ^2^,*N*-Me-Phe ^4^,Gly ^5^-o1]enkephalin (DAMGO), ascorbic acid, salicylic acid, potassium hydroxide, trichloroacetic acid, pyridoxal-5'-phosphate and calmodulin were from Sigma-Aldrich (S. Louis, MO, USA). Tissue Protein Extraction Reagent (T-PER) was obtained by Pierce Biotechnology (Rockford, IL, USA).

### *In vivo *experiments

#### Animals

Male, Wistar rats (200-250 g, Charles River, Monza, Italy) were housed in plastic cages and maintained under controlled conditions with 12-hour light/dark cycles (lights on at 07.00). Tap water and standard laboratory chow were freely available (Additional file 1). It has been demonstrated that the nutrients induce an enhancement of the colorectal sensitivity in both healthy subjects [23] and IBS patients [24,25]. To avoid the influence of the meal on colorectal perception and pain, food was withdrawn 12 hours before surgical procedures and CRD recordings in all *in vivo *experiments [11,12]. However, to verify whether meal could influence the perception of CRD-induced visceral pain, we performed a supplementary experiment on fed rats (n = 5). Experimental procedures were approved by our institutional animal research committees and were in accordance with nationally approved guidelines for the treatment of laboratory animals.

#### Surgical procedure

Rat were anesthetized by an i.p. injection of 70 mg/kg penthotal and were then mounted in a stereotaxic instrument. To perform the i.c.v. injection, a guide cannula (Alzet Brain Infusion Kit II, 3-5 mm) was inserted stereotaxically into the right lateral cerebral ventricle. The stereotaxic coordinates were 1,6 mm right laterally and 0,8 mm dorsoventrally from the bregma and 3,5 mm below the dura. Drugs dissolved in 10 μl saline were injected into the cerebral ventricle by insertion of an injection cannula (28 gauge stainless steel tube) connected to a catheter tube into the guide cannula which was connected to a syringe. In each injection 10 μl of vehicle or drugs were delivered manually into the ventricle over 3 min. At the end of each experiment, methylene blue solution was injected through the injection cannula to verify its correct placement in the right lateral ventricle. Rats exhibiting motor deficits after the surgical procedure were not used in the subsequent experiments.

#### CRD and behavioral testing

All experiments began 1 week after the surgical procedure. Distending procedure were performed as previously described (Additional file 2). The behavioral response to CRD was assessed by measuring the abdominal withdrawal reflex (AWR) as previously described [45,46] (Additional file 2).

#### Effects of H_2_S on colonic nociception

The control group (n = 5) consisted of fasting rats that underwent surgical procedures but not CRD, while the CRD group consisted of fasting rats that underwent surgical procedures and two sets of CRD, the first acting as control. To investigate whether H_2_S administration modulates sensitivity and pain induced by CRD, rats were treated i.p. with vehicle (CRD group) or Na_2_S, an H_2_S donor, at the dose of 100 μMol/kg five minutes before CRD.

#### Effects of the opioid and K_ATP _channels inhibitors

The role of the δ, κ and μ opioid receptors in the H_2_S-induced antinociception was investigated by pre-treating rats with selective opioid receptor antagonists administered at final volume of 10 μl i.c.v.: NTI, a δ opioid receptor antagonist (4 μg/kg), was injected 5 minutes before Na_2_S [47]; GNTI, a κ opioid receptor antagonist (0.08 mg/kg), was administered three days before Na_2_S [48]; CTAP, a μ opioid receptor antagonist (0.09 mg/kg), was administered 30 minutes before Na_2_S [49]. Control experiments were performed by injecting rats with NTI, GNTI and CTAP alone (n = 5 rats/group).

For antisense experiments rats were pretreated with antisense oligodeoxynucleotides direct against specific exons of DOR, KOR and MOR. A mismatched antisense was used as control (Table 1). All antisense olygodeoxynucleotides were administered i.c.v. in dose of 10 μg in 10 μl volume saline [50,52,53]. Treatment with antisenses was performed on day 1, 3 and 5 and the behavioral test was performed at day 6 [51] (Additional file 3).

**Table 1:** Primer used for antisense experiments

Probe sequence	Probe sequence
**DOR-1 opioid receptor clone**	

Exon 1 AS	TGT CCG TCT CCA CCG TGC

Exon 2 AS	ATC AAG TAC TTG GCG CTC TG

Exon 3 AS	AAC ACG CAG ATC TTG GTC AC

**KOR-1 opioid receptor clone**	

Exon 1 AS	GCT GCT GAT CCT CTG AGC CCA

Exon 2 AS	CCA AAG CAT CTG CCA AAG CCA

Exon 3 AS	GGC GCA GGA TCA TCA GGG TGT

**MOR-1 opioid receptor clone**	

Exon 1 AS	CGC CCC AGC CTC TTC CTC T

Exon 2 AS	TTG GTG GCA GTC TTC ATT TTG G

Exon 3 AS	TGA GCA GGT TCT CCC AGT ACC A

Exon 4 AS	GGG CAA TGG AGC AGT TTC TG

**Mismatch**	CGC CCC GAC CTC TTC CCT T

The involvement of K_ATP _channels in the analgesic effects of H_2_S was assessed by pre-treating rats with glibenclamide, a selective K_ATP _channel blocker, at a dose of 2.8 μmol/kg injected intravenously (i.v.) for 20 minutes before Na_2_S administration [11,12] (Additional file 4).

At the end of the CRD procedures, rats were sacrificed and spinal cords (L1-L5) collected for RT-PCR analysis of cFOS [54] (additional file 5) using the following sense and antisense primers: gtctggttccttctatgcag and taggtagtgcagctgggagt.

### *In vitro *experiments

The immortalized human neuronal SKNMCs were used for in *vitro *studies. Cells were grown in Minimum Essential Medium with Earl's salts supplemented with 10% FBS, L-glutamine, penicillin and streptomycin, and regularly passaged to maintain exponential growth.

For *in vitro *studies DAMGO was used at the dose of 1 μM and Na_2_S at the dose of 50 μM. To determine whether H_2_S induces MOR activation, SKNMCs were stimulated with DAMGO or Na_2_S and MOR activation detected by Western blot analysis using a specific antibody raised against a specific epitope in the N-terminus of the receptor that becomes exposed in response to conformational changes induced by receptor activation [55]. This activation-state specific antibody exhibits enhanced recognition of activated receptor [36,37]. In addition, activation of MOR by H_2_S was detected by Western blot analysis of receptor phosphorylation on Serine (Ser) (377). Finally, because MOR activation results in receptor recruitment to β arrestin, co-immunoprecipitation experiments were performed to investigate whether H_2_S induces the formation of a protein-protein complex between MOR and β arrestin (Additional file 6).

#### Effect of H_2_S on MOR internalization

To investigate whether exposure of SKNMCs to H_2_S induces MOR internalization, cells were treated with the μ receptor-selective enkephalin analog DAMGO [15,16] and Na_2_S alone or in combination with the MOR antagonist CTAP. Internalization of the receptor was assessed by Western blot analysis by measuring its translocation from the cell membrane fraction to the cytosol and by confocal microscopy (Nikon) using a specific anti-MOR immunofluorescent antibody (Additional file 7).

#### Effect of H_2_S on AKT phosphorylation

SKNMCs were exposed to DAMGO and Na_2_S up to 60 minutes and Western blot analysis performed on whole cell lysates using a specific antibody that detected the phosphorylated form of AKT on Thre(308). AKT phosphorylation was also detected by measuring the AKT phosphorylated form on Ser(473) (phospho-AKT ELISA KIT, Biosource).

Activation of the PI3K/AKT pathway was tested by exposing SKNMCs to the selective PI3K inhibitor LY294002 (50 μM) in the presence of DAMGO or Na_2_S (Additional file 8).

#### Effect of K_ATP _channels blockade

Expression of K_ATP _channels in SKNMCs was evaluated by assessing the expression of Kir6.2 and SUR1 sub-units (Additional file 9). Qualitative and quantitative PCR were performed by using the following sense and antisense primers: hGAPDH: gaaggtgaaggtcggagt and catgggtggaatcatattggaa; hSUR.1: gtccagatcatgggaggcta and cagaagacagcccctgagac; hKir6.2: gtcaccagcatccactcctt and ggggacttcaaatgttgcat. The effects of glibenclamide (1 μM) on AKT phosphorylation and MOR activation and internalization were determined (Additional file 9).

#### Densitometric analysis

All the densitometric analysis have been performed by using the *Image J *software.

#### Statistical analysis

Behavioral data are presented as mean ± SE, with sample sizes of at least 5 rats per group. Statistical comparisons of unpaired data were performed by the Mann-Whitney test, while statistical comparisons of paired data were performed by the Wilcoxon signed rank test. Densitometric data have been analyzed with Turkey's multiple comparison test. Data on AKT phosphorylation are presented as mean ± SE, with sample sizes of at least 5 experiments per group. An associated probability (p value) of less that 5% was considered significant.

## Competing interests

The authors declare that they have no competing interests.

## Authors' contributions

ED and SF conceived the study and wrote the manuscript. S Cianetti wrote the manuscript. S Cipriani and AM carried out the *in vivo *studies and helped to draft the manuscript (Methods section). BR and MM carried out the *in vitro *studies and helped to draft the manuscript (Methods section). All authors read and approved the final manuscript.

## Supplementary Material

Additional file 1 **Animals**. This file describes the animals used. Click here for file

Additional file 2 **CRD and behavioral testing**. This file describes the behavioral testing used in the *in vivo *studies. Click here for file

Additional file 3 **Effects of the opioid receptors antagonism**. This file describes the methods used for blocking the opioid receptors. Click here for file

Additional file 4 **Effects of K_ATP _channels**. This file describes the method used for blocking the K_ATP _channels. Click here for file

Additional file 5 **Spinal cFOS expression**. This file describes the method used for determining spinal cFos expression. Click here for file

Additional file 6 **Effect of H_2_S on MOR**. This file describes the methods used to determine MOR activation Click here for file

Additional file 7 **Effect of H_2_S on MOR internalization**. This file describes the methods used to detect MOR internalization. Click here for file

Additional file 8 **Effect of H_2_S on AKT phosphorylation**. This file describes the methods used to determine AKT phosphorylation. Click here for file

Additional file 9 **Effects of glibenclamide**. This file describes the methods used to determine the effects of K_ATP _channels blockade. Click here for file
