# Supplementary material for: Hydrogen sulphide induces μ opioid receptor-dependent analgesia in a rodent model of visceral pain
Source: Mol Pain. 2010 Jun 11;6:36. doi: 10.1186/1744-8069-6-36 (PMC2908066; doi:10.1186/1744-8069-6-36)
Supplement: Additional file 1 — Animals. This file describes the animals used. [file 1744-8069-6-36-S1.DOC]

**Additional file 1**

**Animals**

**This file describes the animals used.**

Male, Wistar rats (200-250 g, Charles River, Monza, Italy) were housed in plastic cages and maintained under controlled conditions with 12-hour light/dark cycles (lights on at 07.00). Tap water and standard laboratory chow were freely available. Food was withheld for 12 hours before surgical procedures and CRD recordings. All the animals, including the control and operated (after recovery from surgery) rats were individually trained by spending 2-3 hours per day in a Plexiglas cage for 2-3 days. This allowed them to adjust to a movement-restriction environment similar to that adopted during the distending procedure. All experimental procedures described below were approved by our institutional animal research committees and were in accordance with nationally approved guidelines for the treatment of laboratory animals. All experiments were performed in conscious rats and were conducted in blind manner in that the observer was not aware of the identity of drugs administered to each animal.
