# Supplementary material for: Hydrogen sulphide induces μ opioid receptor-dependent analgesia in a rodent model of visceral pain
Source: Mol Pain. 2010 Jun 11;6:36. doi: 10.1186/1744-8069-6-36 (PMC2908066; doi:10.1186/1744-8069-6-36)
Supplement: Additional file 2 — CRD and behavioral testing. This file describes the behavioral testing used in the in vivo studies. [file 1744-8069-6-36-S2.DOC]

**Additional file 2**

**CRD and behavioral testing**

**This file describes the behavioral testing used in the *in vivo* studies.**

All experiments began 1 week after the surgical procedure. The night before experiments the balloons (7-8 mm diameter) were inflated and left overnight so that the latex stretched and the balloons became compliant. On the testing day, each rat was sedated with ether inhalation and the latex balloon (1.5 cm long) and the probe catheter (0.5 cm) was inserted intrarectally and fixed at the base of the tail. The balloon was connected via a double barreled cannula to a pressure transducer to continuously monitoring the colorectal pressure by a computer (PowerLab PC, A.D. Instruments, Milford, MA, USA) and to a syringe for inflation/deflation of the balloon. The rats were then housed in a small Plexiglas cage (20 x 8 x 8 cm) on an elevated platform and allowed to regain consciousness and adapt for 1 hour. After recovery from sedation, the rats underwent the CRD procedure and behavioral response was tested in all groups except control group in which no CRD was performed. Infusion of water was performed by hands. CRD of 20 seconds performed every 5 minutes was applied in increment of 0.4 ml starting from 0.4 ml and increasing to 1.6 ml water. To achieve an accurate measurement of the colonic parameters and perception, each distension was repeated twice and data were averaged for analysis.

One week after the surgical procedure all rats, except those served as control, underwent a double set of CRD (0.4-1.6 ml water): after 20-30 minutes from the first set of CRD, rats were administered with i.c.v. and/or i.p. drugs and then underwent a second set of CRD (see the next paragraph for details). Behavioral responses and colonic parameters collected during the first and the second sets of CRD were assessed and compared.

The behavioral response to CRD was assessed by measuring the abdominal withdrawal reflex (AWR) using a semiquantitative scoring system [1]. The AWR is an involuntary motor reflex similar to the visceromotor reflex, but it has the great advantage that the latter requires abdominal surgery to implant recording electrodes and wires in the abdominal muscle wall, which may cause additional sensitization [2]. Measurement of the AWR consisted of visual observation of the rat’s response to graded CRD by a blinded observer and assignment of an AWR score according with the behavioral scale previously described [1] in which grade 0 corresponds to no behavioral response to CRD, grade 1 corresponds to brief head movement at the onset of the stimulus followed by immobility, grade 2 corresponds to a mild contraction of abdominal muscles although the rat does not lift the abdomen off the platform, grade 3 corresponds to a strong contraction of the abdominal muscles with the lifting of the abdomen off the platform, and grade 4 corresponds to a severe contraction of the abdominal muscles manifested by body arching and the lifting of the abdomen and of the pelvic structures and scrotum. The rats that did not show any behavioral response (i.e. score 0) were excluded (about 15%). To determine the effect of drugs on colonic smooth muscle, the compliance of the colon during CRD was obtained from colorectal volume and pressure and expressed as ml/mmHg.

**References**

1. Al-Chaer ED, Kawasaki M, Pasricha PJ: **A new model of chronic visceral hypersensitivity in adult rats induced by colon irritation during postnatal development.** *Gastroenterology* 2000, **119**:1276-1285.
2. Ness TJ, Gebhart GF: **Visceral pain: a review of experimental studies.** *Pain* 1990, **41**:167-234.
