# Supplementary material for: Hydrogen sulphide induces μ opioid receptor-dependent analgesia in a rodent model of visceral pain
Source: Mol Pain. 2010 Jun 11;6:36. doi: 10.1186/1744-8069-6-36 (PMC2908066; doi:10.1186/1744-8069-6-36)
Supplement: Additional file 3 — Effects of the opioid receptors antagonism. This file describes the methods used for blocking the opioid receptors. [file 1744-8069-6-36-S3.DOC]

**Additional file 3**

**Effects of the opioid receptors antagonism**

**This file describes the methods used for blocking the opioid receptors.**

The role of the δ, κ and μ opioid receptors on the H_2_S-induced antinociception was studied by testing the effect of pre-treatments with the following selective opioid receptor antagonists: the δ opioid receptor antagonist NTI administered at the dose of 4 μg/kg i.c.v. five minutes before Na_2_S [1], the κ opioid receptor antagonist GNTI administered at the dose of 0.08 mg/kg i.c.v. three days before Na_2_S administration [2] and the μ opioid receptor antagonist CTAP administered at the dose of 0.09 mg/kg i.c.v. thirty minutes before Na_2_S administration [3]. All drugs were dissolved in 10 μl volumes of 0.9% saline. To verify that the listed drugs were not pronociceptive, experiments with NTI, GNTI and CTAP alone were also performed (n=5 rats/group).

To confirm the data obtained with selective opioid receptor antagonists, we performed a second set of experiments by pre-treating rats with antisense oligodeoxynucleotides probes directed against specific exons of the DOR, KOR and MOR. A mismatched antisense oligodeoxynucleotide probe was used as control (Table 1). All antisense olygodeoxynucleotides were administered i.c.v. in 10 µg doses dissolved in 10 µl volumes of 0.9% saline based upon their previously determined effectiveness in agonist-induced feeding studies without producing non-specific effects [4-7]. The chosen antisense oligodeoxynucleotide was administered on day 1, 3 and 5 and the behavioral test was performed at day 6 [5].

At the end of the CRD procedures, rats were sacrificed and blood, colon and spinal cord (L1-L5) were removed and collected for further analysis.

**References**

1. Calcagnetti DJ, Holtzman SG. **Delta Opioid antagonist, naltrindole, selectively blocks analgesia induced by DPDPE but not DAMGO or morphine.** *Pharmacol Biochem Behav* 1991, **38**:185-190.
2. Jewett DC, Grace MK, Jones RM, Billington CJ, Portoghese PS, Levine AS: **The kappa-opioid antagonist GNTI reduces U50,488-, DAMGO-, and deprivation-induced feeding, but not butorphanol- and neuropeptide Y-induced feeding in rats.** *Brain Res* 2001, **909**: 75-80.
3. Sterious SN, Walker EA: **Potency differences for D-Phe-Cys-Tyr-D-Trp-Arg-Thr-Pen-Thr-NH2 as an antagonist of peptide and alkaloid micro-agonists in an antinociception assay**. *J Pharmacol Exp Ther* 2003, **304**: 301-309.
4. Rossi GC, Pan Y-X, Brown GP, Pasternak GW: **Antisense mapping the MOR-1 opioid receptor: evidence of alternative splicing and a novel morphine-6β-glucuronide receptor.** FEBS Letters 1995, **369**: 192-196.
5. Rossi GC, Leventhal L, Pan YX, Cole J, Su W, Bodnar RJ, Pasternak GW: **Antisense mapping of MOR-1 in rats: distinguishing between morphine and morphine-6β-glucoronide antinociception.** *J Pharmacol Exp Ther* 1997, **281;**109-114.
6. Silva RM, Grossman HC, Hadjimarkou MM, Rossi GC, Pasternak GW, Bodnar RJ: **Dynorphin A_1-17_-induced feeding: pharmacological characterization using selective opioid antagonists and antisense probes in rats.** *J Pharmacol Exp Ther*  2002, **301**: 513-518.
7. Israel Y, Kandov Y, Khaimova E, Kest A, Lewis SR, Pasternak GW, Pan YX, Rossi GC, Bodnar RJ: **NPY-induced feeding: pharmacological characterization using selective opioid antagonists and antisense probes in rats.** *Peptides* 2005, **26**:1167- 1175.
