# Supplementary material for: Hydrogen sulphide induces μ opioid receptor-dependent analgesia in a rodent model of visceral pain
Source: Mol Pain. 2010 Jun 11;6:36. doi: 10.1186/1744-8069-6-36 (PMC2908066; doi:10.1186/1744-8069-6-36)
Supplement: Additional file 4 — Effects of KATP channels. This file describes the method used for blocking the KATP channels. [file 1744-8069-6-36-S4.DOC]

**Additional file 4**

**Effects of K_ATP_ channels**

**This file describes the method used for blocking the K_ATP_ channels.**

The involvement of K_ATP_ channels in the modulation of visceral perception by H_2_S was assessed by pre-treating rats with glibenclamide, a selective K_ATP_ channel blocker, at a dose of 2.8 μmol/kg i.v. for 20 minutes before Na_2_S administration (glibenclamide plus Na_2_S group) or glibenclamide alone (glibenclamide group). At the end of the CRD procedures, rats were sacrificed and blood, colon and spinal cord (L1-L5) were collected for further analysis.
