# Supplementary material for: Hydrogen sulphide induces μ opioid receptor-dependent analgesia in a rodent model of visceral pain
Source: Mol Pain. 2010 Jun 11;6:36. doi: 10.1186/1744-8069-6-36 (PMC2908066; doi:10.1186/1744-8069-6-36)
Supplement: Additional file 5 — Spinal cFOS expression. This file describes the method used for determining spinal cFos expression. [file 1744-8069-6-36-S5.DOC]

**Additional file 5**

### **Spinal cFOS expression**

**This file describes the method used for determining spinal cFos expression.**

To determine whether CRD induced nociception, spinal cFOS was detected as a marker of colonic nociception [1]. Total RNA was isolated *in vitro* from rat colon and bone marrow using TRIzol reagent (INVITROGEN). One μg RNA was purified of the genomic DNA by DNaseI treatment (Invitrogen) and random reverse-transcribed with Superscript II (Invitrogen) in 20 μl reaction volume. Fifty ng template was used in 25 µl final volume reaction of Real-Time PCR contained the following reagents: 0.3 µM of each primer and 12.5 µl of 2X DyNAmo SYBR Green qPCR master mix (Finnzymes) . All reactions were performed in triplicate and the thermal cycling conditions were: 2 minutes at 95°C, followed by 40 cycles of 95°C for 20 seconds, 55 °C for 20 seconds and 72°C for 30 seconds in iCycler iQ instrument (Biorad, Hercules, CA).The mean value of the replicates for each sample was calculated and expressed as cycle threshold (C_T_: cycle number at which each PCR reaction reaches a predetermined fluorescence threshold, set within the linear range of all reactions). The amount of gene expression was then calculated as the difference (ΔC_T_) between the C_T_ value of the sample for the target gene and the mean C_T_ value of that sample for the endogenous control (GAPDH). Relative expression was calculated as the difference (ΔΔC_T_) between the ΔC_T_ values of the test sample and of the control sample (WT) for each target gene. The relative quantitation value was expressed and shown as 2^-ΔΔC^_T_. cFos PCR primer was designed using software PRIMER3-OUTPUT using published sequence data from the NCBI database. The sense and antisense sequences were gtctggttccttctatgcag and taggtagtgcagctgggagt respectively.

**Reference**

1. Bonaz B, Rivière PJ, Sinniger V, Pascaud X, Junien JL, Fournet J, Feuerstein C: **Fedotozine, a kappa-opioid agonist, prevents spinal and supra-spinal Fos expression induced by a noxious visceral stimulus in the rat.** *Neurogastroenterol Motil* 2000, **2**:135-147.
