# Supplementary material for: Hydrogen sulphide induces μ opioid receptor-dependent analgesia in a rodent model of visceral pain
Source: Mol Pain. 2010 Jun 11;6:36. doi: 10.1186/1744-8069-6-36 (PMC2908066; doi:10.1186/1744-8069-6-36)
Supplement: Additional file 6 — Effect of H2S on MOR. This file describes the methods used to determine MOR activation [file 1744-8069-6-36-S6.DOC]

**Additional file 6**

## **Effect of H_2_S on MOR**

**This file describes the methods used to determine MOR activation.**

To determine the intracellular mechanisms that regulate the relationship between H_2_S and MOR, we performed several *in vitro* experiments by using the immortalized human neuronal SKNMC cells that were grown at 37°C in Minimum Essential Medium with Earl’s salts supplemented with 10% FBS, L-glutamine, penicillin and streptomycin. Cells were regularly passaged to maintain exponential growth.

To determine whether H_2_S induces MOR activation, we utilized a novel approach that allows to evaluate the activation status of the receptor itself. Antibodies raised against extracellular regions of several GPCRs have been shown to directly modulate their receptor activity [1] and it has been recently demonstrated that activation-state specific antibodies direct toward the N-terminal region of MOR exhibit enhanced recognition of activated receptor [2]. Briefly, SKNMCs serum starved were stimulated with DAMGO (1 μM) and Na_2_S (50 μM) and MOR stimulation with specific antibodies was detected following the manufacturer’s suggested protocol.

Activation of MOR by H_2_S was also detected by studying the receptor phosphorylation on serine 377. Briefly, SKNMCs serum starved were stimulated with Na_2_S at 50 μM or DAMGO at 1 μM for 3, 6, 9, 15 and 30 minutes. After stimulation, total lysates were prepared by solubilization of cells in NuPage sample buffer (Invitrogen) containing Sample reducing agent (Invitrogen) and separated by polyacrylamide gel electrophoresis (PAGE). The proteins were then transferred to nitrocellulose membranes (Bio-Rad) and probed with primary antibodies anti-phospho-MOR (LifeSpan Biosciencies) or anti-MOR (Santa Cruz Biotechnology). The anti-immunoglobulin G horseradish peroxidase conjugate (Bio-Rad) was used as the secondary antibody, and specific protein bands were visualized using Super Signal West Dura (Pierce), following the manufacturer’s suggested protocol.

To study if MOR internalization is due to direct interaction with β arrestin we performed a co-immunoprecipitation experiment. Briefly, SKNMCs serum starved were stimulated with Na_2_S (50 μM) or DAMGO (1 μM) for 5, 15 and 30 minutes. Cells were first washed three times with ice-cold PBS and lysed with E1A lysis buffer with protease inhibitor cocktail (Roche). Lysates were clarified by centrifugation at 13,000*g* for 10 min, and the protein concentration was adjusted to 1 mg/ml. From 1 to 4 mg of total proteins were precleared on a rotating wheel for 1 h at 4°C using protein A Sepharose beads (Amersham Biosciences). Immunoprecipitation was performed overnight at 4°C with 1  μg/ml anti-MOR (Santa Cruz Biotechnology) in the presence of 10 μl of protein A Sepharose (Amersham Biosciences). The resultant immunoprecipitates were washed five times with 1 ml of lysis buffer and then separated by polyacrylamide gel electrophoresis (PAGE). The proteins were then transferred to nitrocellulose membranes (Bio-Rad) and probed with primary antibodies anti-β arrestin (Santa Cruz Biotechnology). The anti-immunoglobulin G horseradish peroxidase conjugate (Bio-Rad) was used as the secondary antibody, and specific protein bands were visualized using Super Signal West Dura (Pierce), following the manufacturer’s suggested protocol.

**References**

1. Gupta A, Décaillot FM, Gomes I, Tkalych O, Heimann AS, Ferro ES, Devi LA. **Conformation state-sensitive antibodies to G-protein-coupled receptors.** *J Biol Chem* 2007, **282**:5116-5124.
2. Gupta A, Rozenfeld R, Gomes I, Raehal KM, Décaillot FM, Bohn LM, Devi LA: **Post-activation-mediated changes in opioid receptors detected by N-terminal antibodies.** *J Biol Chem* 2008, **283**:10735-10744.
