# Supplementary material for: Hydrogen sulphide induces μ opioid receptor-dependent analgesia in a rodent model of visceral pain
Source: Mol Pain. 2010 Jun 11;6:36. doi: 10.1186/1744-8069-6-36 (PMC2908066; doi:10.1186/1744-8069-6-36)
Supplement: Additional file 7 — Effect of H2S on MOR internalization. This file describes the methods used to detect MOR internalization. [file 1744-8069-6-36-S7.DOC]

**Additional file 7**

**Effect of H_2_S on MOR internalization**

**This file describes the methods used to detect MOR internalization.**

To establish that MOR internalization occurs and can be observed in SKNMC cells, we first determined the effect of the μ receptor-selective enkephalin analog DAMGO (1 μM), that it is known to induce μ receptor internalization [1-2], and thereafter we examined the ability of Na_2_S (50 μM) to induce MOR internalization by using both cell membrane fraction techniques and MOR immunofluorescence.

To determine that H_2_S induced MOR internalization and to establish the time-course of this process, cell cultures of SKNMCs were serum starved for 24 hours and then incubated for 5, 15, 30 and 60 minutes with Na_2_S (50 μM) or DAMGO (1 μM). Briefly, plasma membranes were extracted using Qproteome Plasma Membrane Protein Kit (Qiagen) following the manufacturer’s suggested protocol. The extracted proteins were separated by polyacrylamide gel electrophoresis (PAGE), transferred to nitrocellulose membranes (Biorad) and probed with primary antibody MOR (Santa Cruz). The anti-immunoglobulin G horseradish peroxidase conjugate (Bio-Rad) was used as the secondary antibody, and specific protein bands were visualized using Super Signal West Dura (Pierce), following the manufacturer’s suggested protocol. As the maximal effect of H_2_S on MOR internalization was observed at 60 minutes, this time was selected for the further experiments

Cells were fixed in Met-OH / Acetone for 10 minutes and washed three times with ice cold PBS for 5 min. After the pre-incubation in PBS solution containing 3% goat serum for 90 minutes, cells were incubated with the primary antibody anti-MOR (Santa Cruz Biotechnology) diluted in PBS 1% goat serum for 30 min at room temperature. Cells were then washed three times with PBS and incubated with secondary antibody (FITC-goat anti rabbit IGg 62-6111, Zymed) diluted 1:50 in PBS for 45 minutes. Cells were finally washed 3 times in PBS, dehydrated and mounted with Vectashield mounting medium (Vector). The images were acquired with a confocal microscopy. Negative controls included the omission of the primary antibodies or secondary antibody. No staining was observed under the negative control conditions.

**References**

1. Sternini C, Brecha NC, Minnis J, D'Agostino G, Balestra B, Fiori E, Tonini M: **Role of agonist-dependent receptor internalization in the regulation of μ opioid receptors.** *Neuroscience* 2000, **98**: 233-241.
2. Sternini C, Spann M, Anton B, Keith DE Jr, Bunnett NW, von Zastrow M, Evans C, Brecha NC: **Agonist-selective endocytosis of μ-opioid receptor by neurons in vivo.** *Proc Natl Acad Sci* 1996, **93**:9241-9246.
