# Supplementary material for: Hydrogen sulphide induces μ opioid receptor-dependent analgesia in a rodent model of visceral pain
Source: Mol Pain. 2010 Jun 11;6:36. doi: 10.1186/1744-8069-6-36 (PMC2908066; doi:10.1186/1744-8069-6-36)
Supplement: Additional file 8 — Effect of H2S on AKT phosphorylation. This file describes the methods used to determine AKT phosphorylation. [file 1744-8069-6-36-S8.DOC]

**Additional file 8**

**Effect of H_2_S on AKT phosphorylation**

**This file describes the methods used to determine AKT phosphorylation.**

To determine whether H_2_S induced PI3K/AKT pathway activation, the effect of Na_2_S on AKT phosphorylation on threonine 308 residue was detected after time-dependent exposure (up to 60 minutes) of SKNMCs to both DAMGO (1 μM) and Na_2_S (50 μM). Briefly, total lysates from SKNMCs stimulated with Na_2_S or DAMGO for 5, 15, 30 and 60 minutes were separated by polyacrylamide gel electrophoresis (PAGE). The proteins were then transferred to nitrocellulose membranes (Bio-Rad) and probed with primary antibody anti-phospho-AKT (threonin 308) (Cell Signaling). The anti-immunoglobulin G horseradish peroxidase conjugate (Bio-Rad) was used as the secondary antibody, and specific protein bands were visualized using Super Signal West Dura (Pierce), following the manufacturer’s suggested protocol.

To determine the role of the selective MOR receptor inhibition on MOR internalization, the neuronal cells were pre-treated with CTAP at the dose of 1 μM and plasma membrane fraction experiments were conducted after DAMGO (1 μM) and Na_2_S (50 μM) treatments. Furthermore, the effect of CTAP on AKT phosphorylation was also determined. Briefly, SKNMCs were serum starved and then stimulated with both DAMGO (1 μM) and Na_2_S (50 μM) in presence or in absence of CTAP (1 μM) for 60 minutes. After stimulation, total lysates were used for Western blotting (threonin 308) and AKT phosphorylation on serine 473 (phospho-AKT (serine 473) ELISA KIT, Biosource) as previously described.

To determine whether the H_2_S-induced MOR internalization was dependent from PI3K/AKT pathway activation, the selective PI3K inhibitor LY294002 (50 µM) was administered to SKNMC cells and plasma membrane fraction experiments were conducted after DAMGO (1 μM) and Na_2_S (50 μM) treatments. Furthermore, to determine the role of PI3K/AKT pathway on H_2_S-induced AKT phosphorylation on serine 473 (phospho-AKT (serine 473) ELISA KIT, Biosource), the selective PI3K inhibitor LY294002 was added to total lysates from SKNMC cells.
