# Supplementary material for: Hydrogen sulphide induces μ opioid receptor-dependent analgesia in a rodent model of visceral pain
Source: Mol Pain. 2010 Jun 11;6:36. doi: 10.1186/1744-8069-6-36 (PMC2908066; doi:10.1186/1744-8069-6-36)
Supplement: Additional file 9 — Effects of glibenclamide. This file describes the methods used to determine the effects of KATP channels blockade. [file 1744-8069-6-36-S9.DOC]

**Additional file 9**

**Effects of glibenclamide**

**This file describes the methods used to determine the effects of K_ATP_ channels blockade.**

As the K_ATP_ channel antagonist glibenclamide reverses the H_2_S-induced analgesia *in vivo*, we determined whether SKNMCs express the K_ATP_ channels sub-units Kir6.2 and SUR1. Briefly, total RNA from SKNMCs and HepG2 was isolated using the TRIzol reagent according to the manufacturer’s specifications (Invitrogen, Milan, Italy). One μg RNA was purified of the genomic DNA by DNaseI treatment (Invitrogen) and random reverse-transcribed with Superscript II (Invitrogen) in 20 μl reaction volume. Fifty ng template was used in 25 µl final volume reaction of Real-Time PCR as previously described. For qualitative PCR, the amplification of cDNA (50 ng) was achieved in 50 μl mixture containing 200 nM dNTPs, 1.5 mM MgCl_2_, 200 nM of gene specific sense and antisense primers and 1 U of Platinum Taq DNA Polymerase (Invitrogen, Milan, Italy). PCR was conducted as follow: after an initial denaturation at 94°C for 5 minutes , 35 cycles of amplification (94°C for 30 seconds, 58°C for 15 seconds, 72°C for 30 seconds) were performed followed by 5 minutes final extension at 72°C. The quality of RNA samples was evaluated using GAPDH specific primers. PCR products were separated by electrophoresis on 2% agarose gel stained with ethidium bromide 0.5 µg/ml. The band of each target transcript was visualized and photographed by ultraviolet transillumination (Biorad, Gel Doc 2000). Qualitative and quantitative PCR were performed by using these following sense and antisense primers: hGAPDH: gaaggtgaaggtcggagt and catgggtggaatcatattggaa; hSUR.1: gtccagatcatgggaggcta and cagaagacagcccctgagac; hKir6.2: gtcaccagcatccactcctt and ggggacttcaaatgttgcat.

To determine whether glibenclamide reverses the H_2_S-induced MOR activation, SKNMCs serum starved were stimulated with DAMGO (1 μM) and Na_2_S (50 μM) in presence or in absence of glibenclamide (1 μM) for 60 minutes. After stimulation total lysates were separated by polyacrylamide gel electrophoresis (PAGE), transferred to nitrocellulose membranes (Bio-Rad) and probed with primary antibody directed to activated MOR (opioid receptor μ polyclonal antibody - Assay Design). The anti-immunoglobulin G horseradish peroxidase conjugate (Bio-Rad) was used as the secondary antibody, and specific protein bands were visualized using Super Signal West Dura (Pierce), following the manufacturer’s suggested protocol. To determine whether glibenclamide reverses the H_2_S-induced PI3K/AKT activation, total lysates from SKNMCs stimulated with DAMGO (1 μM) and Na_2_S (50 μM) in presence or in absence of glibenclamide (1 μM) for 60 minutes were separated by polyacrylamide gel electrophoresis (PAGE), transferred to nitrocellulose membranes (Bio-Rad) and probed with primary antibodies anti-phospho-AKT (threonin 308) or anti-AKT (Cell Signaling). Furthermore, to determine the effect of H_2_S on AKT phosphorylation on serine 473, in presence of glibenclamide, total lysates from SKNMC cells were assayed by using the phospho-AKT (ser473) ELISA KIT (Biosource) following the manufacturer’s suggested protocol.
